# Negation’s Not Solved: Generalizability Versus Optimizability in Clinical Natural Language Processing

**DOI:** 10.1371/journal.pone.0112774

**Published:** 2014-11-13

**Authors:** Stephen Wu, Timothy Miller, James Masanz, Matt Coarr, Scott Halgrim, David Carrell, Cheryl Clark

**Affiliations:** 1 Department of Health Sciences Research, Mayo Clinic, Rochester, Minnesota, United States of America; 2 Children's Hospital Boston Informatics Program, Harvard Medical School, Boston, Massachusetts, United States of America; 3 Human Language Technology Department, The MITRE Corporation, Bedford, Massachusetts, United States of America; 4 Group Health Research Institute, Seattle, Washington, United States of America; 5 Oregon Health and Science University, Portland, Oregon, United States of America; University Hospitals of Geneva, Switzerland

## Abstract

A review of published work in clinical natural language processing (NLP) may suggest that the negation detection task has been “solved.” This work proposes that an *optimizable* solution does not equal a *generalizable* solution. We introduce a new machine learning-based Polarity Module for detecting negation in clinical text, and extensively compare its performance across domains. Using four manually annotated corpora of clinical text, we show that negation detection performance suffers when there is no in-domain development (for manual methods) or training data (for machine learning-based methods). Various factors (e.g., annotation guidelines, named entity characteristics, the amount of data, and lexical and syntactic context) play a role in making generalizability difficult, but none completely explains the phenomenon. Furthermore, generalizability remains challenging because it is unclear whether to use a single source for accurate data, combine all sources into a single model, or apply domain adaptation methods. The most reliable means to improve negation detection is to manually annotate in-domain training data (or, perhaps, manually modify rules); this is a strategy for optimizing performance, rather than generalizing it. These results suggest a direction for future work in domain-adaptive and task-adaptive methods for clinical NLP.

## Introduction

Negation in unstructured clinical text is a well-known phenomenon. It is crucial for any practical interpretation of clinical text, since negation is common in clinical narrative. For example, the medical significance of “no wheezing” is quite different from that of “wheezing.” With the increasingly widespread use of electronic medical records (EMRs), computational methodologies for negation detection have also become well-known, most notably the early and strikingly straightforward NegEx algorithm [1]. In NegEx, simple regular expressions yield solid performance on detecting the negation of Findings, Diseases, and Mental or Behavioral Dysfunctions from the Unified Medical Language System (UMLS). The success of NegEx (and other techniques) is attributable to the constrained pragmatics of clinical text: because physicians are writing the text in order to convey the health status of a patient, there is a limit to the ways that medically pertinent concepts can be negated. Since existing algorithms have performed well in many published studies [2]–[8], many clinical natural language processing (NLP) practitioners consider negation detection a solved problem (see Table 1′s summary of Related Work) with a simple, generalizable solution.

However, our present work will show that this “solved” designation is premature because current solutions are easily *optimizable* but not necessarily *generalizable*. Negation detection is still a challenge when considered from a practical, multi-corpus perspective, i.e., one in which an algorithm is deployed in many clinical institutions and on many sources of text. For simplicity in this article, we will consider each corpus as its own “domain,” though we recognize that each corpus bridges multiple medical subdomains and all sources that we consider consist only of clinical text.

As the NLP Attribute Discovery team for the Strategic Health IT Advanced Research Project on the Secondary use of the EHR (SHARPn), we attempted to detect negation in four corpora, using machine learning, rules, domain adaptation, and various evaluation scenarios. These corpora include the new SHARPn NLP Seed Corpus of clinical text with multiple layers of syntactic and semantic information, including named entities (NEs) and polarity (i.e., negation). We also used the 2010 i2b2/VA NLP Challenge corpus, the MiPACQ corpus, and the NegEx Test Set. The SHARPn Polarity Module used in our evaluation is currently available in Apache cTAKES (clinical Text Analysis and Knowledge Extraction System; ctakes.apache.org) as part of the ctakes-assertion project, including an integrated domain adaptation algorithm [9]. cTAKES is a comprehensive clinical NLP tool based on the Unstructured Information Management Architecture (UIMA), including (among other things) named entity recognition and negation detection.

We conclude that practical negation detection is not reliable without in-domain training data and/or development. Thus, it can be optimized for a domain, but is difficult to generalize across domains. “Benchmark” gold standard data sets differed sufficiently to have a profound effect on the viability of negation detection algorithms. Furthermore, it is difficult to determine an optimal mix of training data, or to standardize a definitive “benchmark” metric, since both are influenced by corpus-specific annotation guidelines and data sources. The results we report here should remind users of negation detection algorithms to be vigilant in tuning systems to their data, whether by training with local data or modifying rules. We also call for future work in domain-adaptive and task-adaptive methods.

After a discussion of the extensive related work in negation detection, the remainder of this article will introduce the data and methods for corpus and system comparisons of negation detection, present the resulting performance of systems on the different corpora, and discuss implications for negation detection and annotation schema in the larger picture of clinical informatics.

## Related Work

Negation has been studied philosophically since the time of Aristotle; computational efforts addressing negation and related evidentiality/belief state issues have surfaced much more recently [10]. In the clinical domain, negation detection was a very practical early motivation for NLP adoption among the informatics community, and thus significant effort has gone into this task. While there have been many systems implementing negation detection, publicly available corpora for testing them are limited by patient privacy concerns, as is typical in clinical NLP.

Negation detection systems have shown excellent performance in clinical text, beginning with the rule-based NegEx algorithm [1]. NegEx was originally evaluated on spans of text that matched UMLS Findings, Diseases, and Mental or Behavioral Dysfunctions among 1000 test sentences sampled from discharge summaries at the University of Pittsburgh Medical Center; a regression test set was released later with de-identified notes of 6 different types. NegEx has produced numerous updated and customized systems [11], [12], including the updated version released with ConText [13] which performed well on a benchmark NegEx Test Set (available at https://code.google.com/p/negex/wiki/TestSet). Our tests used the YTEX (Yale cTAKES Extensions) version of NegEx [14] as a baseline and included the NegEx Test Set as a benchmark.

Similar to NegEx, many other negation algorithms take a rule-based approach, with a variety of techniques: lexical scan with context free grammar [6], negation ontology [3], or dependency parse rules [7]. Some negation algorithms treat the problem as a machine learning classification task [4] or as some hybrid between rules and machine learning [2], [5]. The performance of these systems and their data sources is summarized in Table 1 below.

**Table 1:** Extensive successful previous work on negation detection in clinical text.

Algorithm	Data source	Entities	Method	Prec.	Rec.	F1
**Negfinder** [6]	10 surgery notes & discharge summaries	UMLS concepts	Lexical/syntax rules	91.84	95.74	92.96
**NegEx** [1]	UPMC ICU discharge summaries	clinical conditions	Trigger/scope rules	84.49	77.84	80.35
**Neg assignment grammar** [3]	Hopkins HNP notes	SNOMED concepts	Negation ontology	91.17	**97.19**	93.90
**Neg. Detection Module** [5]	Stanford radiology reports	unmapped text phrases	Regex/syntax rules	**98.63**	92.58	**94.91**
**ConText** [13]	UPMC 6 note types	clinical conditions	Trigger/scope rules	92	94	93
**MITRE assertion** [2]	2010 i2b2/VA	“problem” phrases	Cue words, CRFs	92	95	94
**DepNeg** [7]	Mayo clinical notes	symptoms & diseases	Dependency path rules	96.65	73.93	83.78

All these general approaches were represented in the 2010 i2b2/VA NLP Challenge task on assertions [8]. In addition to catalyzing innovation from multiple systems, this shared task produced a benchmark data set that is available for research with a simple data use agreement; it interprets negation on medical problem NEs as an assertion that the problem is absent.

The four corpora used in our study all annotate *named entities* explicitly; here, we consider named entities to be spans of text that refer to real-world entities or events that may or may not be classified or mapped to some external ontology. These corpora do not explicitly include the *scope of negation indicators* – i.e., the maximum span within a negation cue word could be applicable.. Some efforts have reversed this, giving an implicit notion of named entities but an explicit notion of negation scope: notably the BioScope Corpus [15] that was used as part of the CoNLL 2010 Shared Task [16]. Bioscope annotates negation, uncertainty, and their scopes on de-identified clinical free text (1,954 radiology reports), biological full articles (9 articles from FlyBase and BMC Bioinformatics), and scientific abstracts (1,273 abstracts also in the GENIA corpus). This is in contrast to the work we present here, which focuses on named entities. We ignore scope for two reasons: First, the lack of gold standard named entity mentions is an additional source of error that no other corpus would have, making the comparison unfair. Second, while negation scope annotations overcome some recall issues for non-standard terminology (e.g., “patient is not feeling as much like a pariah today” would represent negation correctly despite finding no NE), they do not overcome issues in fine-grained annotation guideline distinctions (see Section 3.3 on Annotation Guidelines).

## Methods

Here, we first describe the annotated NLP corpora used in training and testing, with salient information about the gold standard entity and negation annotation guidelines. We then describe the new SHARPn Polarity Module and the YTEX NegEx rule-based baseline.

### 3.1 Ethics statement

We did not seek IRB approval as all the data used in this study were collected from previous studies. While the data sets were from electronic medical records that originally included protected health information, all medical records were reliably de-identified before we had access to the data sets. Thus, none of the authors had access to any patient identifying information. Three of the corpora (the SHARPn corpus, MiPACQ corpus, and i2b2 corpus) were available to us with signed Data Use Agreements between the supplier and recipient institutions. One (the NegEx Test Set) was freely downloadable online with no restrictions.

### 3.2 NLP corpora with negation annotations

Our work used four clinical NLP annotation efforts; the SHARPn NLP Seed Corpus, the 2010 i2b2/VA NLP Challenge Corpus; the MiPACQ corpus; and the NegEx Test Set. Statistics in Table 2 show their overall relative sizes, train/test splits, and proportion of negated concepts.

**Table 2:** Characteristics of four corpora with negation annotations.

	sharp		i2b2		mipacq		negex
	Train	Test	Train	Test	Train	Test	Test
**Num. Documents**	140	22	349	477	2,443	324	120
**Num. Sentences**	5,014	569	33,022°	48,482°	19,672	2,236	2,376*
**Num. Named Entities**	10,575	1,154	11,968	18,550	23,249	1,721	2,371
**Num. Negated NEs**	918	48	2,535	3,609	1,681	158	491
**% Negated NEs**	8.7%	4.2%	21.2%	19.5%	7.2%	9.2%	20.7%
**Data Source(s)**	Mayo, Group Health		Partners, BIDMC, UPMC		Medpedia, NLM ClinQ, Mayo		UPMC

*subset selected manually; °automatic sentence detection on pre-whitespace-tokenized text.

First, the SHARPn NLP Seed Corpus consists of de-identified radiology notes related to Peripheral Arterial Disease (PAD) from Mayo Clinic, and de-identified breast oncology progress notes regarding incident breast cancer patients from Group Health Cooperative. This multi-layered annotated corpus follows community adopted standards and conventions for the majority of annotation layers, which include syntactic trees, predicate-argument structure, coreference, UMLS named entities, UMLS relations, and Clinical Element Models (CEM) templates [17]. Negation is included in the CEM templates as an attribute of UMLS concepts.

Second, the 2010 i2b2/VA NLP Challenge Corpus contained manually annotated, de-identified reports from Partners Healthcare, Beth Israel Deaconess Medical Center, and the University of Pittsburgh Medical Center. The majority of notes were discharge summaries, but the University of Pittsburgh Medical Center also contributed progress notes.

Third, the MiPACQ corpus [18], [19] annotates multiple syntactic and semantic layers, similar to the SHARPn NLP corpus. There are three major divisions to the sources of data: a snapshot of *Medpedia articles* on medical topics, written by clinicians, retrieved on April 26, 2010; *clinical questions* from the National Library of Medicine’s Clinical Questions corpus (http://clinques.nlm.nih.gov), collected by interviews with physicians; and sentences from Mayo Clinic clinical notes and pathology notes related to colon cancer.

Finally, the NegEx Test Set is a set of manually-selected sentences from 120 de-identified University of Pittsburgh Medical Center reports (20 each of radiology, emergency department, surgical pathology, echocardiogram, operative procedures, and discharge summaries). This set was used to evaluate the ConText algorithm [13], while another 120 reports of similar distribution (not publically available) were used for the development of the negation portion of ConText (i.e., an updated NegEx).

### 3.3 Comparison of annotation guidelines

Manually annotated negation in one of these corpora is not strictly equivalent to that in other corpora. We cannot directly compare annotation guidelines because we do not have corpora that are *multiply-annotated* with different guidelines. However, we should note that all annotation projects reported high inter-annotator agreement within their respective projects. Here, we qualitatively analyze the annotation guidelines concerning the annotation of both NEs (concepts) and attributes (assertion status), hypothesizing that some differences in annotation guidelines may negatively affect the performance of negation algorithms across corpora.

The primary difference between the annotation guidelines of the corpora appears to be in the definition of NEs, rather than direct indications of how negation should be handled. First, NE annotation guidelines differ in the *semantic types that are allowed*. The broadest is the MiPACQ corpus, which annotates 17 UMLS Semantic Groups. (However, in practice, some semantic groups have zero or negligible frequencies, and we have grouped them together in our analysis.) SHARP only annotates the 6 most clinically relevant groups, namely, Diseases and Disorders, Signs and Symptoms, Labs, Medications, Procedures, and Anatomical Sites. These semantic group divisions and their respective distributions are enumerated in Table 3, for these two corpora. The NegEx Test Set is much more narrow, including only Signs, Symptoms, Diseases, and Findings (but not differentiating between these) with qualitative values. The i2b2 corpus is similarly restrictive, only annotating “problems,” i.e., Diseases, Signs and Symptoms. Thus, they are excluded from Table 3.

**Table 3:** In the MiPACQ and SHARP corpora, the named entities (NEs) are annotated with different semantic groups which occur with different frequencies (left columns).

	NEs by semantic group, percentage(number)								Negated NEs, percentage(number)							
	SHARPn				MiPACQ				SHARPn				MiPACQ			
	Train		Test		Train		Test		Train		Test		Train		Test	
**AnatomicalSite**	20.36%	(4591)	25.24%	(428)	39.87%	(4216)	50.69%	(585)	4.01%	(184)	7.48%	(32)	0.43%	(18)	–	
**DiseaseDisorder**	26.53%	(5981)	23.29%	(395)	27.54%	(2912)	29.29%	(338)	7.82%	(468)	11.65%	(46)	17.07%	(497)	13.31%	(45)
**Lab**	–		–		1.91%	(202)	0.69%	(8)	–		–		2.97%	(6)	25.00%	(2)
**Medication**	14.74%	(3324)	13.50%	(229)	2.98%	(315)	–		4.60%	(153)	8.30%	(19)	6.35%	(20)	–	
**Procedure**	19.62%	(4424)	22.52%	(382)	16.64%	(1759)	11.01%	(127)	3.28%	(145)	1.05%	(4)	3.01%	(53)	–	
**SignSymptom**	16.28%	(3671)	12.68%	(215)	5.70%	(603)	2.17%	(25)	19.83%	(728)	26.51%	(57)	52.57%	(317)	4.00%	(1)
**Entity**	0.35%	(79)	0.06%	(1)	2.96%	(313)	3.73%	(43)	1.27%	(1)	–		0.64%	(2)	–	
**Event**	2.10%	(474)	2.71%	(46)	2.40%	(254)	2.43%	(28)	0.42%	(2)	–		4.42%	(5)	–	

The prevalence of Negated NEs also differs by corpus and semantic group (right columns).

The corpora also differ in the *span to consider* when identifying NEs. NegEx Test Set is the most permissive, annotating whole clinically-relevant phrases as NEs regardless of their syntactic type (e.g., the statement “Right ventricular function is normal” is treated as a single entity as shown by the underlining). i2b2/VA guidelines only consider whole noun and adjective phrases as possible NEs (e.g., “her shortness of breath and coughing resolved” includes the modifier “her” in the NE). Similar to i2b2/VA, MiPACQ also indicates that whole noun phrases should be candidate NEs, but smaller units are typically used in practice (e.g., “her chest x-ray” leaves out the modifier “her”). SHARP predominantly annotates maximal strings that match UMLS terms as NEs, which often excludes long paraphrases and closed-class modifying adjectives (similar to MiPACQ), although there are some cases of CUI-less NEs and multi-span NEs.

Another difference in NE annotation guidelines is the *amount of overlap allowed* between NEs. The NegEx Test Set has only one phrase annotated per sentence, hence no overlap in NEs; i2b2/VA only annotates full noun and adjective phrases, so fully subsumed NEs are not allowed. In contrast, SHARP annotates subspans as long as they are mapped from the UMLS and of a different semantic type (e.g., both “chest” (anatomical site) and “chest x-ray” (procedure) in “her chest x-ray”). MiPACQ removes this restriction of different semantic types, but stipulates that some relationship must be shared between the subspan and the full span – this is in practice very similar to SHARP (e.g., there is a locationOf relationship between “chest” and “chest x-ray”).

Overall, the four guidelines are not as precise with negation annotation definitions as they are with NEs. The SHARP, MiPACQ, and NegEx Test Set representations imply a relation between an explicit negation marker and the negated term (e.g., a cue word like “no” would be marked, and the following term “shortness of breath” would then set a negation_indicator = present). The i2b2/VA guideline assumes a pragmatic inference about the intent of the author in describing his/her observations (e.g., “no shortness of breath” would mark assertion = absent without marking the cue word). This difference does lead to some minor morphology-related annotation differences. For example, “afebrile” is marked as “absent” for i2b2, but not in SHARP, MiPACQ, or NegEx Test Set since there is no external negation indicator.

### 3.4 SHARPn Polarity Module

As with many existing approaches, the SHARPn Polarity module treats negation detection as a classification problem for NEs. We engineered features that would make sense of the context surrounding an NE:

- **Token in Bag-of-Words (BOW).** These most basic, binary features indicated whether a given word appeared within a window (bag) from the NE. For example, one feature might be whether “no” occurred in the 5 preceding words. We included several different BOWs, based on directionality (preceding vs. following the NE) and size (3, 5, or 10).
- **Token in positional context.** These features are similar to BOW features, but are specific to the exact position with respect to the NE of interest (e.g., “without” occurred 4 words preceding the NE). Windows of 4 and 5 were considered.
- **Cue words.** Following MITRE’s successful negation detection system [2], we identified cue words – an expert-curated list of negation-related words (e.g., “negative for”). The nearest cue word in scope and its category (a normalized word or phrase, e.g., “negative”) were included as binary features.
- **Dependency path rules.** We directly utilized the rule-based DepNeg system [7] to produce binary features corresponding to whether the NE lay along a dependency path that typically specifies negation. For example, “no evidence of coughing, rales, or wheezing” has “wheezing” outside a 5-word window, but is connected by a dependency parse path to “no.”
- **Constituency tree fragments.** In addition to dependency path rules, we also used constituency tree fragments. The constituent parser within cTAKES is Ratnaparkhi’s Maximum Entropy parser [20] as implemented in OpenNLP, trained on clinical treebanks. Tree fragments (partial constituency trees) can represent, for example, that the NE in question sits inside an adjective phrase “negative for <concept>.” Fragments are automatically extracted and defined following Pighin and Moschitti [21]; training data determines whether the features are useful or not.

Examples of these features are included in a table in the Discussion section. The size of the feature set is upper-bounded by the size of the training set’s vocabulary and diversity of tree fragments; there are 12 dependency path rules. In practice, a feature vector, **v,** will be smaller than this upper bound, since not every dictionary word is in the context of an NE.

The SHARPn Polarity Module classifies each NE based on these features. We chose to utilize classifiers via ClearTK because of its compatibility with UIMA-based systems like cTAKES [22]. After some preliminary experimentation with various classifiers, we selected linear kernel SVMs implemented with LIBLINEAR, which learn decision boundaries (negated vs. not negated) based on the distribution of features in the training data. SVMs are considered to have good generalization performance due to inherent regularization, and excel in situations (like ours) where there are a massive number of features. Since linear kernel SVMs require only one parameter to be tuned, we manually tuned it during development using cross-validation.

Training data for a single model can consist of more multiple corpora. In a standard setting, instances from different corpora would not be differentiated during training. Alternatively, we implemented an optional domain adaptation algorithm, frustratingly easy domain adaptation (FEDA) [9], to build some of our multi-corpus models. FEDA is a simple but effective domain adaptation technique that requires in-domain training data. If there are data from four domains *a, b, c,* and *d*, for example, a model would be trained with 5 concatenated (row) feature vectors: **v** = [**v** *_all_* **v** *_a_* **v** *_b_* **v** *_c_* **v** *_d_* ]. A training sample from domain *a* will be logged in **v** *_all_* and **v** *_a_* only, whereas a training sample from from domain *b* will be logged in **v** *_all_* and **v** *_b_* only, and so forth. At test time, the domain of the test sample is supplied to the classifier, and instances are classified with a weighting of the domain-specific model in concert with the “general” model.

### 3.5 Evaluation Setup

Our evaluations used the NegEx algorithm as a baseline, as implemented in the Yale cTAKES Extensions (YTEX) [14]. Using Named Entities discovered by the standard cTAKES pipeline, the YTEX negation module set the “polarity” attribute of each NE to −1 (negated) or +1 (not negated). Because NegEx is a rule-based method, we would expect it to be immune to performance improvement or degradation based on training data. However, it is well-known that customization of rules is likely necessary when applying NegEx in settings other than the one in which it was initially developed [11], [12].

**Figure 1:**
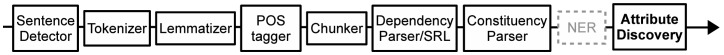
The cTAKES Pipeline. The SHARPn Polarity Module is an Attribute Discovery algorithm. Training and evaluations use gold standard NEs (skip NER).

The SHARPn Polarity module was implemented within the cTAKES system (see Figure 1), leveraging feature extraction and machine learning programming interfaces available in the ClearTK suite of tools (available at https://code.google.com/p/cleartk/). It should be noted that we did preliminary tests using χ^2^ feature selection (filtering out the feature if their χ^2^ values were too low), but the performance did not significantly improve. Thus, we have left feature selection out of the results of this study; some sample χ^2^ values for specific features are listed in the Discussion section. The polarity module used in our tests is currently available as a tagged branch of the Apache cTAKES source code repository, and will be part of a future cTAKES release.

For both training and testing, we used gold standard NEs and negation annotations as defined in each of the corpora. System negation annotations are compared to gold standard for precision, recall, and F-measure (the harmonic mean of precision and recall). We also used the default cTAKES pipeline to produce anything besides NEs or negation annotations (e.g., sentence annotations, tokens, POS tags, dependency parses, constituency parses, semantic role labels; see ). While there is some risk for error propagation from these other components into negation detection, we believe this risk is minimized for the main precision, recall, and F-measure metrics, because systemic errors would appear in both training and testing data, and any impact on negation performance would be mediated through their representation in a machine learning feature vector.

We trained the SHARPn Polarity module on each of the four corpora; train/test splits were provided for the SHARPn, i2b2/VA, and MiPACQ corpora; for these three corpora, training and testing in our evaluations uniformly respected these training and testing splits (e.g., even in cases like training on SHARP data but testing on i2b2 data). Because the NegEx Test Set’s corresponding development set was not available, we used the NegEx Test Set in any single evaluation as either the training data or the testing data. The tables presenting our results use parantheses to show when reusing training data invalidates the test performance measures (i.e., training and testing would have been on the same data).

## Results

### 4.1 Single test corpus performance

**Figure 2:**
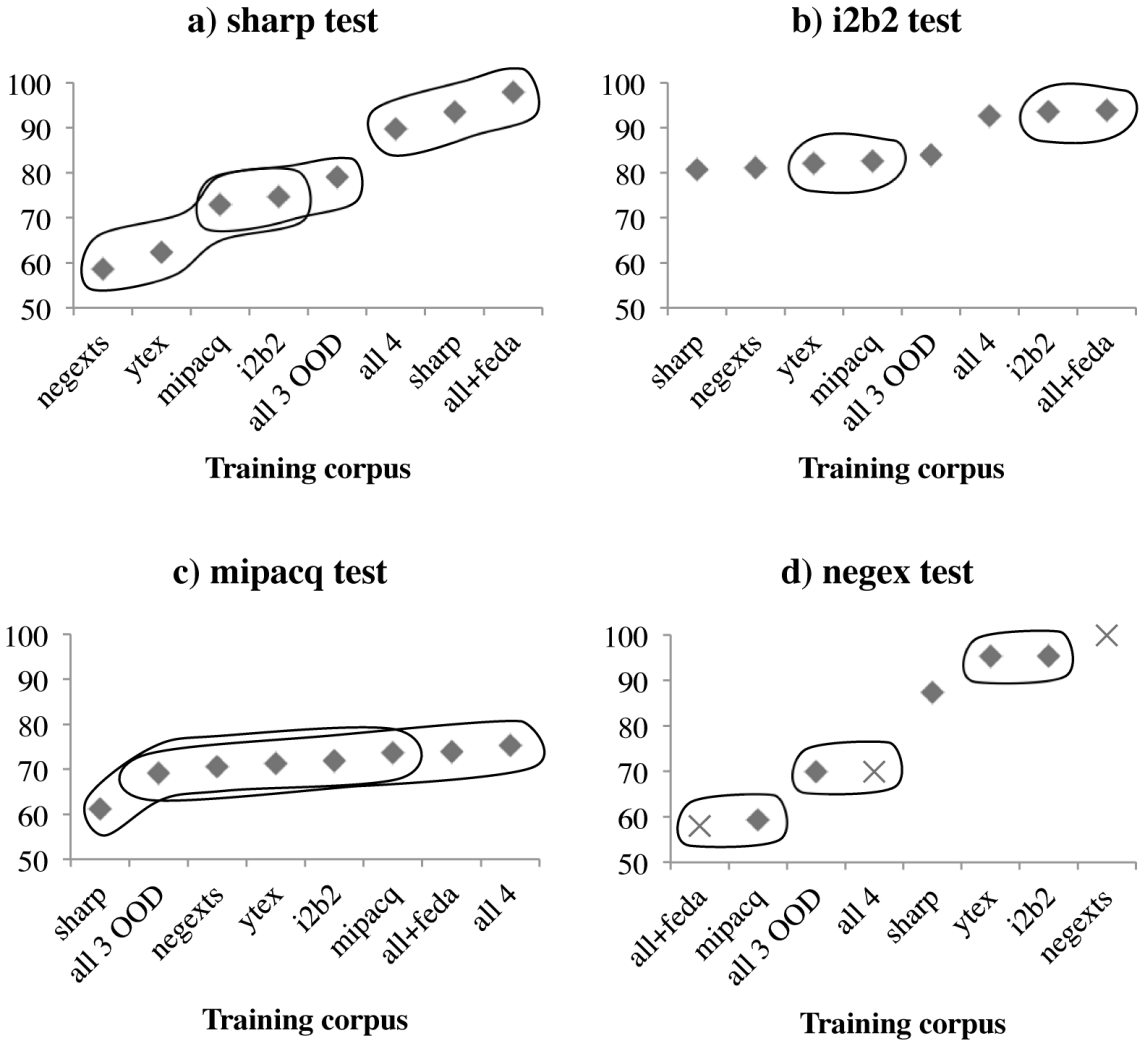
Significance bands of model performance for each test corpus. These are labeled with successive letters from right to left in Table 4.

The practical question a user might ask is: “How can I maximize negation detection performance for my data?” Table 4 below illustrates the difficulty of answering this question by showing performance on four corpora (columns) by various systems (rows). Row 0 gives previously reported comparison statistics for i2b2 data (MITRE [2]) and the NegEx TestSet (GenNegEx 1.2.0, see https://code.google.com/p/negex/wiki/TestSet); SHARP and MiPACQ do not have previous results to compare with. We have grouped these systems to be representative of three strategies for negation detection that are used in the community: the unedited, rule-based YTEX algorithm (row 1); machine learning classifiers when only out-of-domain data (OOD) is available (rows 2–6); and machine learning classifiers when some in-domain data is available (rows 7–9). Note that row 7 is equivalent to the diagonal from rows 2–6, namely, where the training set and test set are from (different portions of) the same corpus. Table 4 also includes significance bands down each column; pair-wise approximate randomization significance tests for F_1_ score, aggregated by document, are reported for *p<*0.05. Values in a column labeled with different successive superscripted letters (e.g., 93.9^a^ and 92.6^b^) indicate that there is a significant difference between two systems. These bands are further visualized in Figure 2.

**Table 4:** Performance (F_1_ score) in practical negation detection situations.

	Test	sharp	i2b2	mipacq	negexts
**Previous**	**0. (various)**	–	94	–	94.6
**Rule-based**	**1. ytex (rules)**	62.3^c^	82.1^d^	71.3^a,b^	95.3^a^
**ML with out-of-domain (OOD) training**	**2. sharp**		80.7^e^	61.2^b^	87.3^b^
	**3. i2b2**	74.7^b,c^		**71.9^a,b^**	**95.4^a^**
	**4. mipacq**	72.9^b,c^	82.6^d^		59.3^d^
	**5. negexts**	58.6^c^	81.1^e^	70.6^a,b^	
	**6. All 3 OOD**	**79.0^b^**	**83.9^c^**	69.1^a,b^	69.9^c^
**ML with in-domain training**	**7. 1 In-Domain**	93.5^a^	93.6^a^	73.6^a,b^	(99.9)
	**8. All 4 corpora**	89.7^a^	92.6^b^	**75.3^a^**	(69.9^c^)
	**9. All+FEDA**	**97.9^a^**	**93.9^a^**	73.9^a^	(58.0^d^)

First, YTEX (top row), implementing the widely used rule-based NegEx algorithm, performed quite well on the NegEx Test Set (F_1_ = 95.3%). When used without modification on other corpora, performance fell to unacceptable levels (e.g., F_1_ = 62.3% on SHARP data). As might be expected, we may conclude that widely-used rule-based algorithms need to be modified according to their target data.

For situations in which only OOD data is available (common in clinical text), one strategy is to use a single OOD corpus as training data (rows 2–5). Using a single OOD corpus has widely varying results, with models ranging from 59.3% to 95.4% F-score on the NegEx Test Set. Another strategy is to “use all the (OOD) data you have” (row 6), but again the results are mixed. With the highest OOD models in bold, it is not clear which strategy is optimal, and it is difficult to tell what pairs of corpora yield good performance. Underlying reasons for this variability are further explored in Section 4.2.

The situation is much improved when in-domain data is available (rows 7–9, with most scores lying within the highest significance band, labeled with superscript ‘a’). Only in MiPACQ data, for which the test set is small, are there OOD models in the same significance bands (i.e., superscript ‘a’ in rows 2–6) as the best models with in-domain data. With in-domain models, we still face the same problem of whether to use a single in-domain corpus (row 7) or to “use all the data you have” (row 8). Only in i2b2 data are improvements statistically significant, and which approach performs better appears to differ by corpus. It may be the case that, since i2b2 data is only on ‘problems,’ including training data from other sources decreases performance; the MiPACQ corpus, being the most general, appears to benefit from training on other corpora.

Using domain adaptation (row 9) is also not conclusively better than a single in-domain corpus (row 7) or leaving out domain adaptation (row 8), since improvements are not statistically significant at the *p*<0.05 level (all share ‘a’ superscripts). Recall that these “All+FEDA” tests (row 9) will train a model with a feature space approximately 5 times the size of the “All” feature spaces (row 8). Without conclusive evidence, it is difficult to say whether the additional model complexity is worth it.

Thus, whether there is in-domain data available or not, we cannot conclude a uniform policy such as “use all available data to train your model” or “train a model on a single most similar corpus” or “always use domain adaptation if possible.” However, we can conclude that annotating in-domain data is the best way to ensure solid performance on a machine learning system. Note that, this is a method of optimizing the performance for a corpus, rather than generalizing performance between corpora.

### 4.2 Corpus difficulty and usefulness

Rather than trying to define an arbitrary scientific measure of corpus ‘similarity,” we consider the practical perspectives of corpus “difficulty” (scores on testing, down columns) and “usefulness” (scores on training, along rows). As evidenced by the OOD rows 2–5 of Table 4, the difficulty and usefulness of corpora seem to vary. Testing on MiPACQ data has an average F_1_ score of 70.9% down the column of trained systems, indicating it is probably the most difficult to test on. Training on i2b2 data (row 3) achieved a macro-averaged F_1_ score of 80.7% across the row of test sets, indicating its training set is perhaps the single most useful for training.

Difficulty and usefulness are not symmetric: i2b2 data is clearly the best OOD training data for the NegEx Test Set (F_1_ = 95.4% in column 4); but NegEx is not the best OOD training data for the i2b2 test set (F_1_ = 81.1% in column 2; MiPACQ is significantly better with F_1_ = 82.6%). These variations in difficulty and usefulness could hypothetically be explained by several factors. For example, the diversity of source data in the MiPACQ corpus (including non-clinical data such as Medpedia) may contribute to its difficulty; MiPACQ in-domain performance is loosely comparable to the OOD performance of other models. Additionally, Section 4.4 below explores differences in the annotation guidelines (as expressed in NE length and semantic group). Different corpora have fundamentally different characteristics, and more samples from one corpus are not equal to those from another.

We also sought determine whether usefulness could be explained by corpus size, hypothesizing that more data would lead to more robust machine learning models. Thus we performed experiments in which the amount of training data was varied. These experiments focus on the i2b2 training data which had a small but consistent advantage in cross-domain experiments. We built learning curves in which we tested on the SHARP Seed, MiPACQ, and i2b2 test sets. We randomly sampled from 10% to 100% of the training data, at increments of 10%. For each sampled proportion size we averaged across 5 runs to compute F-scores at that point.

**Figure 3:**
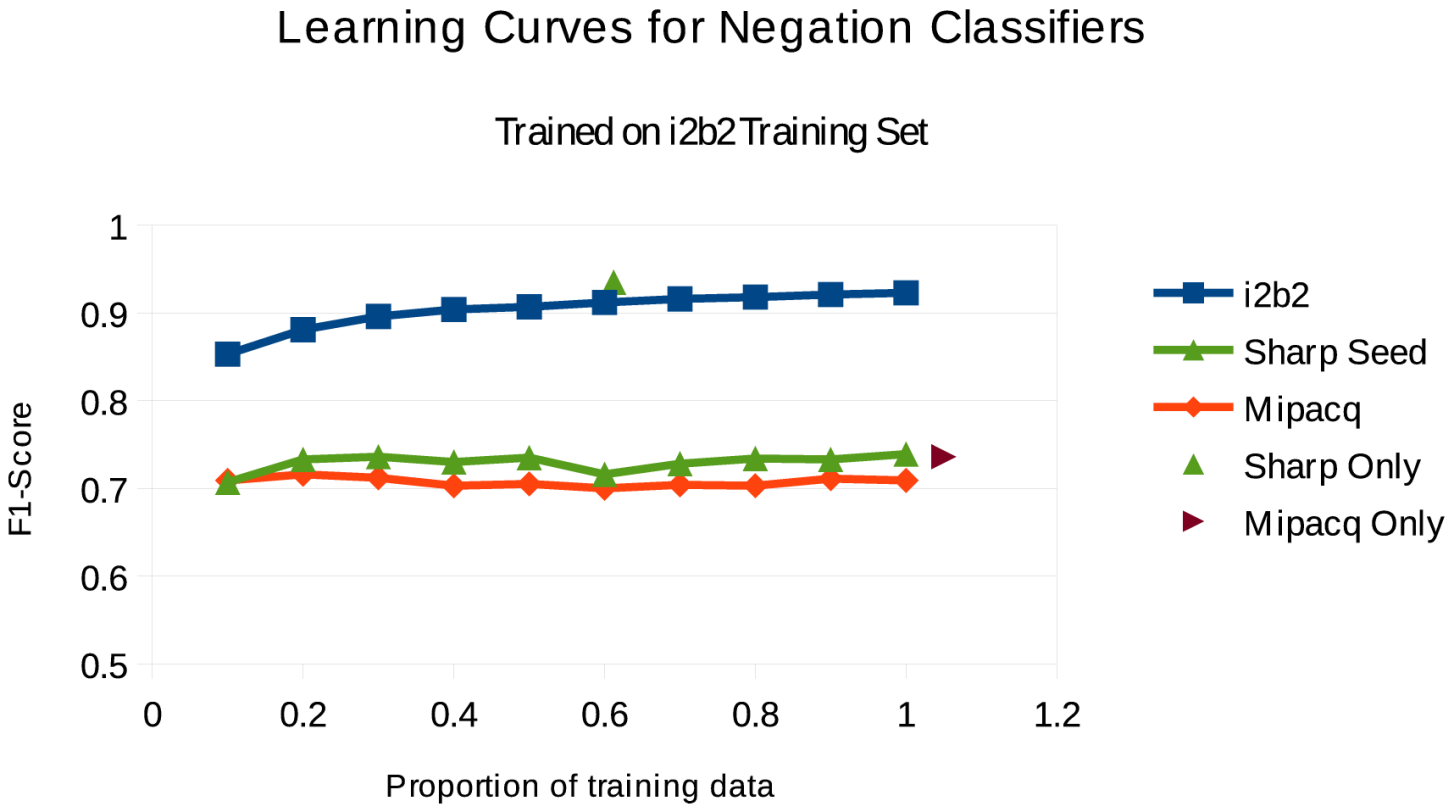
Learning curve for i2b2 training data on various corpora. For each proportion of the i2b2 corpus (x axis), the reported F-score (y axis) is an average of 5 randomly sampled runs.

The results are shown in Figure 3. The learning curve for the i2b2 data seems to be increasing even until the very end, as the classifier seems to be making marginal improvements with ever more data. In contrast, in both cross-domain experiments the performance levels off very early, conservatively estimated at around 20% of the i2b2 training data being used. For additional reference, we have also plotted two points taken from Table 4– the in-domain performance for SHARP and Mipacq. The x-axis for each of these points is the size of the training data (counted as the number of instances of negation), while the y-axis is the F-score obtained on each corpus' in-domain evaluation.

These experiments seem to indicate that the value of the i2b2 corpus is not simply because of its size. In fact, performance on outside corpora of a system trained on 20% of the i2b2 data is comparable to one trained on 100%.

We should be careful to not overstate the distinctions of “most difficult” or “most useful.” Furthermore, overall “usefulness” does not necessarily imply usefulness in a specific OOD setting or corpus, for example, supplementing in-domain training data with the “useful” corpus. In further testing on the SHARP corpus, we considered whether the “useful” i2b2 training data could augment the SHARP training data, and found that adding i2b2 training data did not improve performance on the SHARP corpus (F_1_ = 90.9%), whereas adding MiPACQ did improve performance (F_1_ = 94.6%). Though it is difficult to define “similarity,” it may be the case that more similar corpora can be mixed as training data more effectively.

### 4.3 Average performance

We considered average performance of several models on multiple corpora. In Table 5 we include averages with and without FEDA (i.e., for rows 8–9 of Table 4), labeling pairwise statistical significance at *p*<0.05 between the domain adapted and non-domain adapted versions with an asterisk. The NegEx Test Set is used for training rather than testing.

**Table 5:** Average F-score with and without frustratingly easy domain adaptation (FEDA).

*Test \Train*	All	+ FEDA
**sharp**	89.66	97.87
**i2b2**	92.57	93.93*
**mipacq**	75.29	73.93
**negex**	–	–
**macro-avg**	85.84	88.58
**micro-avg**	91.91	93.28*

Here, we report both macro-averages (arithmetic mean of the three test sets) and micro-averages (weighted by the number of instances in each test set). The micro-averaged scores are heavily weighted towards the i2b2 numbers because the i2b2 test set is the largest; macro-averages, on the other hand, are much lower than has been previously reported in literature, in large part due to the difficulty of the MiPACQ corpus. Overall, i2b2 is the only corpus on which domain-adapted models clearly outperform un-adapted models.

### 4.4 Named Entity characteristics

**Figure 4:**
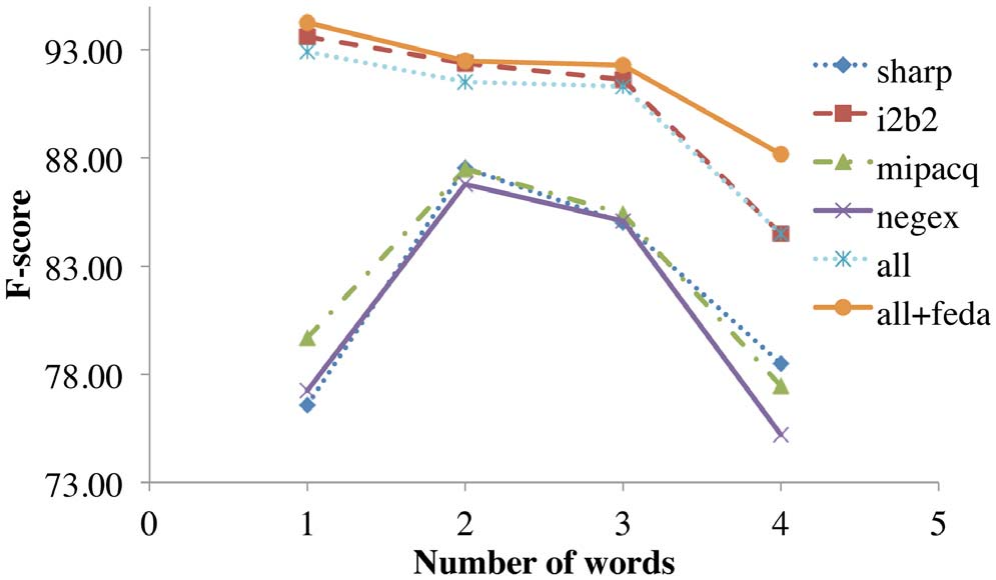
The effect of named entity length (in number of words) on performance for each of 6 training configurations. SHARP, MiPACQ, and i2b2 test sets are used for evaluation.

Negation predictions were further analyzed to see if the differences in NE annotation guidelines influenced performance, since resulting differences in “gold standard” training data could confuse machine learning systems. Because guidelines for annotating NEs differed in how much of a noun phrase to include, we examined NE length in words. Figure 4 shows that the i2b2-trained model has the best overall performance, likely due to its larger number of training samples rather than its similarity to other annotation guidelines. Underscoring this, the NegEx Test Set is the most permissive guideline (allowing whole phrases), yet it obtains similar performance to the restrictive SHARP and MiPACQ guidelines (typically short phrases).

Figure 4 also shows that longer Named Entities are more difficult to negate correctly in all of the corpora; in the i2b2 corpus, single-word terms were easy to negate, whereas in other corpora single-word terms were substantially harder. One hypothesis is that this could be due to i2b2’s different accounting of inherently negated terms such as “afebrile.” “Afebrile” itself accounted for 124 of 3,609 negated NEs in the i2b2 training set, and the number of single-term entities inherently negated by virtue of negative suffixation or negative acronym component (e.g., “NAD” standing for “no acute distress”) total 299 (8.3%). While this does not account for the total error difference in one-word NEs, it is a factor worth noting. Additional annotation differences may result from differing assumptions regarding explicit and implicit expression of negations. Further accounting of these terms may require re-annotation of the corpus, which is out of the scope of this article.

**Figure 5:**
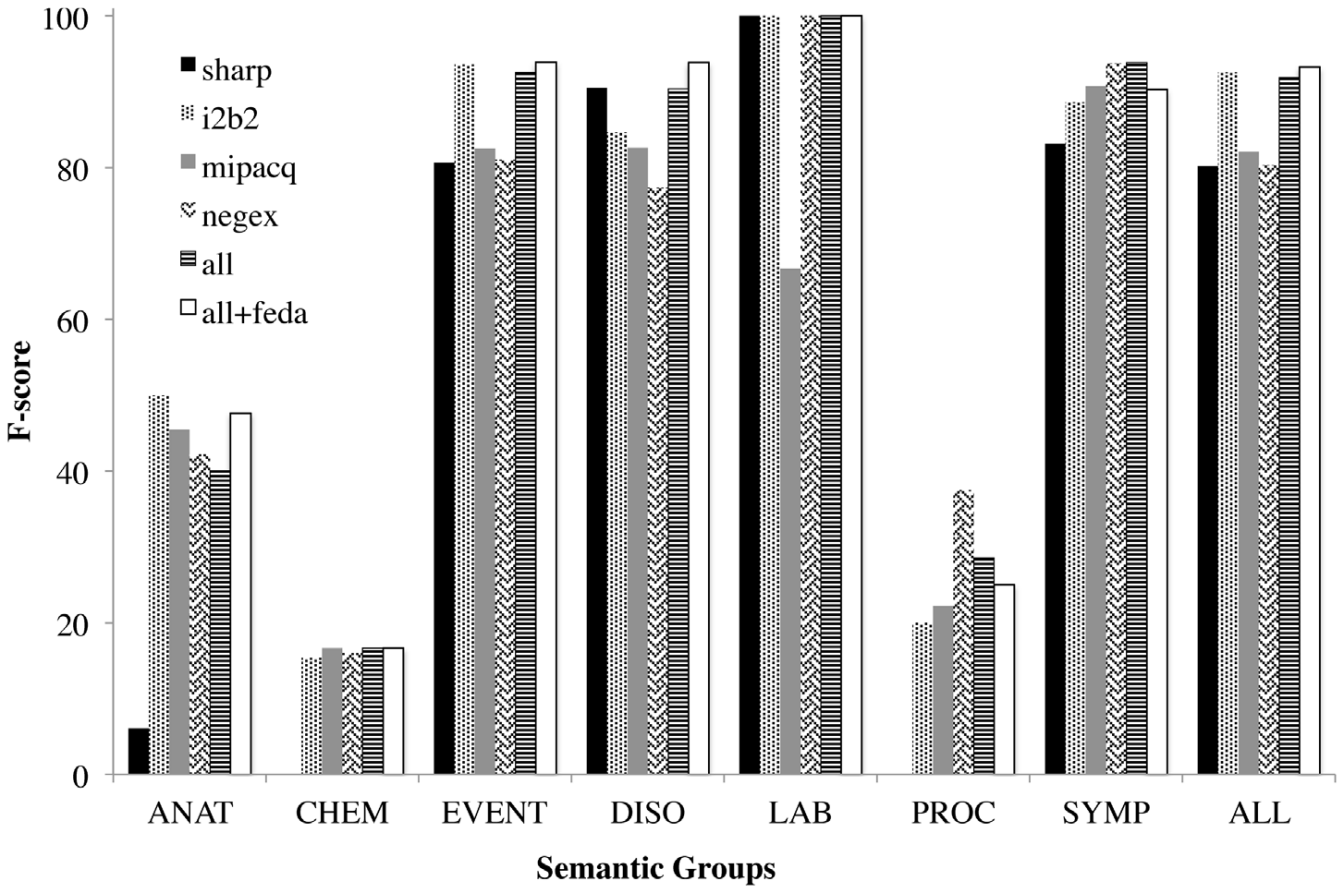
The effect of named entity semantic group on the F-score of 6 models. SHARP, MiPACQ, and i2b2 test sets are used for evaluation.

Because the annotation guidelines also differed in which semantic groups to annotate, we considered performance of each model for each specific semantic group, shown in Figure 5. Recall from Table 3 that SHARP and MiPACQ included a broad selection of semantic groups, including anatomical sites (ANAT), chemicals and drugs (CHEM), disorders (DISO), laboratories (LAB), procedures (PROC), and symptoms (SYMP). i2b2 and the NegEx Test Set only specified “problems” and are considered EVENT in Figure 5.

Despite their annotation guideline similarity, we did not find that SHARP and MiPACQ performed similarly on individual semantic groups. Note in particular the relatively low SHARP-trained performance on ANAT, CHEM, PROC, and SYMP despite its having training data in those groups. A MiPACQ-trained model also did not outperform other models, despite that most of the test set NEs of minority semantic groups came from the MiPACQ corpus. Similarly, the i2b2-trained and NegEx Test Set-trained models had similar annotation guidelines, but did not perform similarly on groups such as EVENT, DISO, or PROC. These models were not uniformly worse than SHARP or MiPACQ on the semantic groups for which they had no training data.

## Discussion

### 5.1 Salient features

From the foregoing tests, NE properties like length and semantic group (and thus, annotation guidelines) did not fully explain the discrepancy in performance between different models. Thus, we qualitatively examined the broader differences between corpora by looking at negation contexts in each corpus. We defined negation contexts as the features of the SHARPn Polarity Module, as defined in Section 3.4.

Table 6 calculates and ranks the χ^2^ statistic corresponding to each feature (i.e., on a 2×2 grid of whether the NE was negated vs. whether the feature was present) within all four sets of training data. Thus, the ranking in Table 6 corresponds to the model trained on “All” training sets, in row 8 of Table 4 and in the preceding section. Table 6 also compares the rank of features in the “all” model to salient features in each individual corpus.

**Table 6:** Top negation context features in a multi-corpus model, by chi-square value; and feature rank in domain-specific models.

		Feature Rank in Training Data				
Feature Description	Chi∧2	all	i2b2	mipacq	negexts	sharp
(D) DepNeg path: dt_nmod_mod	16713.1	1	5	1	2	1
(A) Bag of 5 preceding words: no	15601.1	2	1	3	3	2
(E) Tree Fragment Context Above-Left: (DT no)	15263.2	3	3	2	4	3
(A) Bag of 10 preceding words: no	14928.9	4	2	4	1	5
(A) Bag of 3 preceding words: no	14207.4	5	4	5	5	4
(B) Preceding word #0: no	10683.5	6	6	9	6	10
(C) Cue category: no	9848.3	7	7	6	12	6
(C) Cue word: no	8866.9	8	9	7	7	15
(C) Cue phrase (any negation)	8110.7	9	10	8	8	9
(E) Tree Fragment Context Above-Left:(NP (DT no) (CONCEPT ))	8038.3	10	8	18	13	23
(D) DepNeg path: negverb->dobj_mod	3817.3	11	12	13	16	285
(E) Tree Fragment Context Above-Left:(VBZ semclass_deny)	3809.4	12	13	10	15	851
(A) Bag of 10 preceding words: denies	3081.2	13	22	12	21	1195
(E) Tree Fragment Context Above-Left: (DT any)	2721.2	14	43	11	24	486
(B) Preceding word #2: no	2672.9	15	15	28	22	53
(C) Cue category: deny	2479.0	16	16	19	38	327
(A) Bag of 5 preceding words: denies	2380.3	17	28	16	26	2070
(A) Bag of 5 following words: or	2350.9	18	25	30	9	46
(E) Tree Fragment Context Above-Left: (NP (DT no) (NML ))	2247.9	19	27	44	34	19
(A) Bag of 10 following words: or	2242.1	20	26	29	10	39

Feature types are classified as in Section 3.4.

It is evident that the most important features were consistent across all the corpora, representing the “easy cases” of negation: namely, when the word “no” is related to a concept by proximity or by syntax. The SHARP corpus differs somewhat, likely due to the sources of data for the SHARPn Seed Corpus: Mayo Clinic radiology reports (do not directly report a patient interaction) and Seattle Group Health breast cancer-related notes (only one example of a patient “denying” smoking). This distinction does not explain why MiPACQ, rather than SHARP, is a more “difficult” corpus.

### 5.4 The Big Picture for Negation Detection

Because of the relatively constrained pragmatic uses of negation in clinical text, negation detection algorithms are easy to optimize for specific corpora, as illustrated in Table 1. However, we believe the research community has at times conflated this with being immediately effective off-the-shelf. Evaluation of systems is artificially inflated by the ad hoc development of training and testing corpora and their differing annotation guidelines. When in-domain, consistently-annotated training data is scarce or nonexistent, negation detection performance remains unimpressive (middle portion of Table 4), just as in other NLP problems like parsing or named entity recognition. Furthermore, it is difficult to simply characterize the differences between domains, e.g., by NE length (Figure 4), semantic group (Figure 5) or lexical and syntactic context (Table 6).

To ensure excellent negation performance for a machine learning model, it appears that we still need to annotate examples of negation on the target corpus for fully supervised training (or domain adaptation). Similarly, rule-based methods need a development set and experts who can develop domain-specific rules. Thus, we conjecture that negation is not “solved” until negation is tailored to specific applications and use cases, or until the more general problem of semi-supervised domain adaptation is solved.

## Conclusion

While a review of published work may suggest that the negation detection task in clinical NLP has been “solved,” our multi-corpus analysis of negation detection indicates that it is easy to *optimize* for a single corpus but not to *generalize* to arbitrary clinical text. Though negation detection can be straightforward in constrained settings, both rule-based and machine-learning approaches have mixed results in heterogeneous corpora. Furthermore, more training data was not necessarily better for the common case in which no in-domain data is available. The most significant difference in performance was the availability of in-domain training data, which is inherently a strategy for optimizing performance rather than generalizing it. Furthermore, training on all available data and using domain adaptation techniques did not uniformly benefit performance in a significant way. Future work includes task-adaptive negation detection algorithms and semi-supervised domain adaptation.
